# Meat safety legislation and its opportunities and hurdles for innovative approaches: A review

**DOI:** 10.1016/j.foodcont.2022.109160

**Published:** 2022-11

**Authors:** Gunvor Elise Nagel-Alne, Emil Murphy, Brittany McCauslin, Sigrun J. Hauge, Dorte Lene Schrøder-Petersen, Janne Holthe, Ole Alvseike

**Affiliations:** a Animalia - Norwegian Meat and Poultry Research Center, P.O. Box 396, Økern, N-0513, Oslo, Norway; b Deer Industry New Zealand, PO Box 10701, Wellington, 6140, New Zealand; c BX Foods Oamaru, PO Box 50, Oamaru, 9400, New Zealand; d Danish Technological Institute, Gregersensvej 9, 2630, Taastrup, Denmark

**Keywords:** Legislation, Innovation friendly, Slaughter, Red meat

## Abstract

Albert Einstein has been quoted “We cannot solve our problems with the same thinking we used when we created them”. Innovations are necessary to meet future challenges regarding sustainability, animal welfare, slaughter hygiene, meat safety and quality, not at least for optimal balance between these dimensions. The red meat safety legislation texts from Europe, New Zealand, USA, and global guidelines, were analysed for normative formulations (“how it is or should be done”) that may create non-intentional hurdles to innovation and new technology. Detailed descriptions of slaughtering techniques and meat processes may hinder innovative processing from being investigated and implemented. The identified problematic normative phrases typically either conserve conventional technologies or organisation of the work, prescribe solutions where no established method, objective criteria or limits exits, or put forward visions impossible to obtain. The Codex Alimentarius was found to have less normative formulations and more functional demands (“what to achieve”) than the national and regional regulations. European, New Zealand's and US′ legislation share many similarities and challenges, and they all reflect the prevailing processing methods. Consequences are briefly commented, and alternative objective functional demands suggested. Normative legislation texts provide familiar context easier to understand, but also make legislation voluminous. This review underlines the mutual dependency between risk-based legislation and conditional flexibility, and between functional demands and control activities targeted on measurable objective criteria. The legislation does not have to be either or. Objective normative phrases in legislation can function as a least common multiple if alternative methods are allowed on condition that they fulfil objective criteria. Context and practical advice should mainly come from textbooks, consultants, white papers and in Food Business Operator's own guidelines, among others.

## 1 Introduction

To ensure good hygiene during the slaughtering and processing of meat, legislation allows, prescribes, prohibits, and sets targets for abattoirs and cutting plants. These laws and administrative procedures are given within international, regional, or national jurisdictions. The legislation is guided and supported by principles, codes, guidelines, and agreements, from global institutions such as FAO, WHO, OIE, WTO and the Codex Alimentarius. EU member states and other European countries with trade agreements with the EU, must adapt EU laws and regulations into their national legislation. Correspondingly, the New Zealand legislation has incorporated bilateral agreements with Australia. In USA, federal laws apply to interstate trade and as *de-facto* minimum standards. State and local laws can adopt or modify, but not lessen the stringency, of federal requirements. The competent authorities (CA) are responsible for the implementation of these general principles and international legislation by adaptation to local legal and political structures, traditions, and culture (Codex Alimentarius, 2020). Every country's law and administrative procedures contain provisions that Food Business Operator's (FBOs) must comply with, for example, requirements in regulations for production of safe food should be based on hazard analysis conducted by the companies (Codex Alimentarius, 2020). Further, the General Principles of Food Hygiene from Codex Alimentarius states that a fundamental question for each FBO in every case is “what is necessary and appropriate to ensure the safety and suitability of food for consumption?”.

**Fig. 1:**
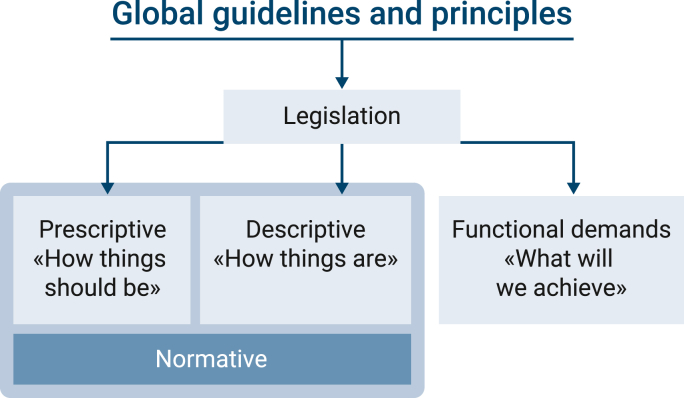
Outline of the connection between global guidelines and principles, development of legislation and the distinction between normative text and functional demands in legislative text.

Legislation is the link between global principles and guidelines and operative food production. Most legislative phrases can be divided into *descriptive* or *prescriptive* texts. Descriptive formulations describe “how things are” and prescriptive formulations describe “how things ought to be”. An example of descriptive text is “post-mortem inspection is taking place after evisceration of the carcasses, the pluck and bowl and intestines are presented in parallel” (European Commission, 2004a). A prescriptive text is “to avoid contamination, they (FBO) must ensure separation in space or time …” (European Commission, 2004a). Another term, *normative* formulations, are used both in law, science, economics, and philosophy. The philosopher David Hume (1711–1776) drew the line between normative and descriptive assertions when he criticized contemporary moral philosophy for drawing normative conclusions from descriptive premises, which he believed did not constitute logically valid conclusions (https://snl.no/David_Hume). The term normative is being used slightly differently by professions, and a perception is that normative is synonymous with prescriptive and expresses an evaluation with a moral dimension, that something is good or bad, right or wrong, relative to a norm or standard (Leftwich, 2015). In this review, we use normative as both descriptive and prescriptive (Fig. 1).

Innovation can be described as the creation, development and implementation of a new product, process or service, with the aim of improving efficiency, effectiveness or competitive advantage (Taylor, 2017). A legal text with detailed description of the sequence and content of production, such as “FBOs … have slaughter lines …” (European Commission, 2004a) will restrict innovative methods where slaughtering is not along a slaughter line. It is the FBO's responsibility to provide safe food, and several systems and solutions are adapted by FBOs to achieve this. Technological solutions do not normally need approval from food safety competent authorities, as it is the FBOs' opportunity and investment risk to find effective solutions for conventional foods. However, when a legislative text is descriptive or prescriptive, such phrases may reduce the freedom to develop new or different systems as a normative text provide limitations in stating “this is how it is or should be done”; i.e., normative phrases may occlude new technologies and disruptive changes. Innovation and development of new systems for dressing of an animals' body into a carcass and other edible and inedible parts, may for instance conflict with current legislation. This reflects the need for what we call “functional demands*”* with an objective and measurable description of *what to achieve* (aim) rather than *how to achieve* it (method) when developing legislative texts. This is in line with the philosophy behind quality assurance systems and Codex’ code (Codex Alimentarius, 2005). The code defines “dressing” functionally as “the progressive separation of the body of an animal into a carcass and other edible and nonedible parts”. While a prescriptive formulation can be morally right but practically impossible, for example “To avoid contamination”, a functional demand would express a maximum acceptance level, measured by standardised methods.

One of the General Principles in Codex Alimentarius (Codex Alimentarius, 2020) states that a food hygiene system should be reviewed periodically and whenever there is a significant change in the system, such as when new scientific knowledge is available, or a request of implementing new equipment or a new process is presented.

Policymakers may consider that rules or legislation should be prescriptive, meaning that the rules clearly prescribe the expected outcome or the wanted direction of development. However, quite often the objects may comply with the rules, but still the conditions or actions do not achieve what was expected. On the opposite, disruptive or innovative systems such as the Meat Factory Cell concept (Alvseike, 2016; Esper, From, & Mason, 2021), where slaughtering is performed in a “cell” instead of along a slaughtering line, may fulfil legislator's intentions better than conventional solutions but still not fully comply with the legislation. Therefore, objective criteria and control of functionality are needed. This approach requires transparent surveillance or control of measurable traits (Blagojevic et al., 2021).

The aim of this review was to assess current red meat safety legislation, guidelines and principles for unnecessary hurdles and opportunities for innovation in the process from lairage to dressing of an animals’ body into edible parts.

## 2 Materials and methods

### 2.1 Text analysis of relevant legislation

A text analysis of different legislative systems was conducted, where legislation concerning red meat was included and not wild game nor farmed game. The scope was the process from lairage to dressing of an animals’ body into edible parts. The legislation was analysed for normative formulations that may make non-intentional hurdles to suppliers, end-users and authorities. Identified issues with normative formulations were listed and alternative functional demands were suggested with scientific references where available. The legislation texts from four jurisdictional perspectives were studied i) worldwide according to Codex Alimentarius and FAO, ii) EU legislation in Europe/EEA, iii) legislation in New Zealand and iv) legislation in USA.

#### 2.1.1 Worldwide guidelines and principles

The Codex Alimentarius Commissions' Code of Hygiene Practice for Meat chapter 8–10 (CAC/RCP 58–2005) and the Codex Alimentarius General Principles of Food Hygiene (CAC/CXC 1–1969), FAO report Perspectives and guidelines on food legislation, with a new model food law (Vapnek & Spreij, 2005), and FAO Technical Guidance Principles of Risk-Based Meat Inspection and their Application (FAO, 2019) were included for analysis. The WHO's draft Global strategy for food safety was also included (WHO, 2022).

#### 2.1.2 Europe and European Union legislation

The hierarchy of EU legislation is laws, regulations that are directly translated into national legislation, directives that are implemented into national legislations’ structures and national or regional specific legislation.

Legislative texts analysed in this study included the following regulations:

1. **2017/625** Regulation (EU) 2017/625 of the European Parliament and of the Council of March 15, 2017 on official controls and other official activities performed to ensure the application of food and feed law, rules on animal health and welfare, plant health and plant protection products (European Commission, 2017).
2. **2019/624** Commission Delegated Regulation (EU) 2019/624 of February 8, 2019 concerning specific rules for the performance of official controls on the production of meat and for production and relaying areas of live bivalve molluscs in accordance with Regulation (EU) 2017/625 of the European Parliament and of the Council (European Commission, 2019a).
3. **2019/627** Commission Implementing Regulation (EU) 2019/627 of March 15, 2019 laying down uniform practical arrangements for the performance of official controls on products of animal origin intended for human consumption in accordance with Regulation (EU) 2017/625 of the European Parliament and of the Council and amending Commission Regulation (EC) No 2074/2005 as regards official controls (European Commission, 2019b).
4. **852/2004** Regulation (EC) No 852/2004 of the European Parliament and of the Council of April 29, 2004 on the hygiene of foodstuffs (European Commission, 2004b).
5. **853/2004** Regulation (EC) No 853/2004 of the European Parliament and of the Council of April 29, 2004 laying down specific hygiene rules for food of animal origin (European Commission, 2004a).

#### 2.1.3 New Zealand (and Australia) legislation

New Zealand laws are generally structured in Acts of Parliament (statues) and secondary legislation. Statues are made by the New Zealand Parliament whereas secondary legislation is law made by someone other than Parliament but under a power that Parliament has delegated to them. As an adherent of Codex Alimentarius, New Zealand food safety statues tend to be focused on outcomes and administrative processes where secondary legislation provides specific technical standards to meet.

The review of legislative text from New Zealand included the following pieces of secondary legislation that are relevant to the production of meat for domestic consumption:

1. Animal Products Notice: Specifications for Products Intended for Human Consumption (New Zealand Government, 2020, pp. 1–115).
2. Animal Products Notice: Ante-mortem and Post-mortem Examination of Mammals, Ostrich and Emu Intended for Human Consumption (New Zealand Government, 2015).

#### 2.1.4 US legislation

For the purposes of this paper, only federal level legislation was in the scope. A bill is introduced to Congress, passed as an Act, and if approved by the president, it becomes a Law. The appropriate regulatory agencies enforce laws through rules, regulations, and policies. Regulations are developed by a public process that involves notice and comments. Federal Government regulatory publications: The federal government publishes the U.S. Code (U. S. C.), which includes the laws coded by subject, Federal Register (FR)- published every weekday, carries all proposed and finalised regulations, legal notices, and executive orders. Code of Federal Regulations (CFR) are rules and regulations grouped by subject areas (“Titles”). Legislative text from CFR, Title 9 (Animals and Animal Products) was analysed in this paper, in particular parts 310 through 329, and part 416 (https://www.ecfr.gov/current/title-9). The government also issue directives; however, these are considered guidance and were not included in this review.

## 3 Results

The results from the legislative text analysis are presented in separate tables according to jurisdictions, with Table 1, Table 2, Table 3, Table 4 presenting worldwide guidelines and principles, EU legislation, New Zealand legislation and USA legislation, respectively. The legislative text analysis is presented with paragraph ID, text in paragraph, identified normative formulations, suggested new formulations and references where applicable.

**Table 1:** Results from text analysis of *CAC/RCP 58–2005 Code of Hygiene Practice for meat* with detection of normative formulations possibly hampering innovative approaches in the handling of food producing animals from lairage to processing a carcass into edible parts.

Paragraph ID	Paragraph text	Identified normative formulations	Suggested alternative functional demand	Reference
Codex Alimentarius CAC/RCP 58–2005 8.3 66. Design and construction of slaughter areas	Where slaughter is carried out, the processing line should be designed so that there is constant progress of animals in a manner that does not cause cross-contamination.	Conventional methodology conserved by prescriptive hygiene legislation.	Either delete “line” or exchange “processing line” with neutral “workflow”.	Alvseike, Prieto, Bjørnstad, and Mason (2020)
		The “processing line” is not a prerequisite for slaughter and can make restrictions when new systems are developed.		Barbier et al. (2020)
Codex Alimentarius CAC/RCP 58–2005 9.4 Hygiene requirements for slaughter and dressing	During dressing, and with due consideration to minimizing contamination: - where bodies of animals are skinned, this process should be completed before evisceration	Conventional methodology conserved by prescriptive hygiene legislation. In some cases, the skin or hide protects the carcass, e.g., emergency slaughter in the field. Also, cross-contamination to deskinned parts is a challenge, and alternative approaches may show hygienic benefits from e.g., delayed deskinning.	The result of applied methods and procedures during dressing and meat inspection must fulfil objective functional demands e.g microbiological criteria and visual contaminants.	Skúladóttir et al. (2022)
				(Livsmedelsverket, 2021a)
				(Svenska Köttföretagen, 2020)
				(Alvseike et al., 2020) (NolChiri Intact Meat Limited, 2018)
				UECBV (2017)
				Hassan, Skjerve, Bergh, and Nesbakken (2015)
				Arguello, Álvarez-Ordoñez, Carvajal, Rubio, and Prieto (2013)
				Greig et al. (2012)
				Desmarchelier, Fegan, Smale, and Small (2007)
				Brown et al. (2000)
Codex Alimentarius CAC/RCP 58–2005 9.7151 Hygiene requirements for process control after post-mortem inspection	When fresh meat is cut or de-boned pre-rigor: - it should be transported directly from the dressing area to the cutting up or de-boning room - the cutting up or de-boning room should be temperature-controlled and directly linked to the dressing area, unless the competent authority approves alternative procedures that provide an equivalent level of hygiene; and - cutting up, de-boning and packing should be done without delay and should meet all requirements for hygienic process control.	Conventional methodology conserved by prescriptive hygiene legislation. The formulation assumes that meat must be cut, or that de-boning must occur in a *different room* than dressing procedures.	The combination of time and temperature during fresh meat production must fulfil objective functional demands.	Troy (2006) Sumner and Krist (2002) Alvseike et al. (2020) Brown et al. (2000)

**Table 2:** Result from text analysis of EU legislation Regulation (EC) No 853/2004, Regulation (EU) 2017/625, Commission Delegated Regulation (EU) 2019/624, Commission Implementing Regulation (EU) 2019/627 and Commission Regulation (EC) No 2074/2005 with detection of normative formulations possibly hampering innovative approaches in the handling of food producing animals from lairage to processing a carcass into edible parts.

Paragraph ID	Paragraph text	Identified normative formulation	Suggested new formulation	Scientific ref.
853/2004 Annex II Section II 2. (d)	The procedures must guarantee that each animal or, where appropriate, each lot of animals accepted onto the slaughterhouse premises: (d) is clean	A normative expectation that FBOs cannot meet. “Clean” is not objectively defined nor accurately measurable. Both structural systematic differences, codes and assessors' judgement of cleanliness can vary and possibly induce unfair assessments and competition. The animal welfare dimension is not addressed clearly.	The FBO must take into account the condition and cleanliness of animals in the lairage, so that the results meet objective functional hygiene and animal welfare demands.	(Dragan Antic, Houf, Michalopoulou, & Blagojevic, 2021) (Livsmedelsverket, 2021a) (Svenska Köttföretagen, 2020) (Animalia & Nortura, 2017) Omer et al. (2015) Hauge et al. (2012) Hauge, Nafstad, Skjerve, Røtterud, and Nesbakken (2011) (D Antic et al., 2010) McEvoy et al. (2000) (Biss & Hathaway, 1996) (Biss & Hathaway, 1995)
853/2004 Annex III Section I Chapter II: Requirements for slaughterhouses 2 (c)	To avoid contamination, they (FBO) must ensure separation in space or time of the following operations: (i) stunning and bleeding; (ii) in the case of porcine animals, scalding, depilation, scraping and singeing; (iii) evisceration and further dressing; (iv) handling clean guts and tripe; (v) preparation and cleaning of other offal, particularly the handling of skinned heads if it does not take place at the slaughter line; (vi) packaging offal; and (vii) dispatching meat;	Conventional methodology conserved by descriptive hygiene legislation. The process of slaughtering and dressing of an animal's body is expected to be undertaken along a slaughter line system with constant progress.	The FBO must have procedures and verification that the slaughtering process fulfil objective functional hygiene demands e.g., microbiological criteria, visual contamination.	Hedman, Andersson, et al. (2021) Hedman, Veggeland, Vågsholm, and Berg (2021) (Dragan Antic et al., 2021) (Dragan Antic et al., 2015) Brown et al. (2000)
853/2004 Annex III, Section I Chapter II: Requirements for slaughterhouses 2 (d) and (e)	(e) have slaughter lines (where operated) that are designed to allow constant progress of the slaughter process and to avoid cross-contamination between the different parts of the slaughter line. Where more than one slaughter line is operated in the same premises, there must be adequate separation of the lines to prevent cross-contamination.	Conventional methodology conserved by prescriptive hygiene legislation. The process of slaughtering and dressing of an animal's body is expected to be undertaken along a slaughter line system with constant progress.	The FBO must have procedures and verification that the slaughtering process fulfil objective functional hygiene demands e.g., microbiological criteria, visual contamination.	Brown et al. (2000)
853/2004 Annex I, Section I, Chapter II, D 1	Post mortem inspection is taking place after evisceration of the carcasses, the pluck and bowl and intestines are presented in parallel. In many abattoirs, one inspector controls the carcass presented on a conveyor and another inspector controls the internal organs on a different conveyor. All external surfaces are to be viewed.	Conventional methodology conserved by descriptive hygiene legislation. There may be alternatives and ideally, PM should be performed by one inspector doing a holistic assessment. The most important hazards are normally not visible.	Carcass and relevant parts of the animal must be appropriately available for PM inspection. Gap: Validation and calibration of PM examinations is highly needed. Harmonised MI codes are needed, regular statistical analysis of results is needed to detect and manage biases. Gap: Reporting systems from MI are many places manual, poor and unable to detect considerable biases.	Alban, Petersen, et al. (2021) Alban, Vieira-Pinto et al. (2021) Antunović et al. (2021) Alvseike et al. (2021) Buncic, Alban, and Blagojevic (2019) Alvseike, Prieto, Torkveen, Ruud, and Nesbakken (2018) (de Freitas Costa, Corbellini, da Silva, & Nauta, 2017) Devitt et al. (2016) Bækbo, Petersen, Larsen, and Alban (2016) Berthe, Hugas, and Makela (2013)
853/2004 Annex III, Chapter III: Requirements for cutting plants	Food business operators must ensure that cutting plants handling meat of domestic ungulates: 1. are constructed so as to avoid contamination of meat, in particular by: (a) allowing constant progress of the operations; or (b) ensuring separation between the different production batches.	Conventional methodology conserved by prescriptive hygiene legislation. The legislation prescribes that cutting and deboning should be performed in specialised plants separated from the operations in the abattoir. “Constant progress” is not necessarily hygienic optimal. Contamination is not avoided by “constant progress”, but bacterial growth will be reduced by reduced Time*Temperature exposure.	The FBO must have procedures and verification that the processing fulfil objective functional hygiene demands e.g., microbiological criteria, visual contamination.	Alvseike et al. (2018) Brown et al. (2000)
853/2004 Annex III, Chapter IV: Slaughter hygiene 7 (c)	Measures must be taken to prevent the spillage of digestive tract content during and after evisceration and to ensure that evisceration is completed as soon as possible after stunning.	Conventional methodology conserved by prescriptive hygiene legislation. “To ensure that evisceration is completed as soon as possible after stunning” is prescriptive and is not a functional objective criterium. The potential problems arising from delayed evisceration are - gas production in intestines that make hygienic evisceration more difficult, - or bacterial growth through intestines, - or deviating sensory characteristics - and onset of rigor mortis	Evisceration should ensure that meat complies with objective functional hygiene demands and should be completed before the meat is damaged due to gas production in intestines, bacterial growth through intestines, deviating characteristics or rigor mortis can occur.	Peruzy et al. (2022) Gill, Penney, and Nottingham (1976) Gill, Penney, and Nottingham (1978)
853/2004 Annex III, Chapter V: Hygiene during cutting and boning 1.	Carcasses of domestic ungulates may be cut into half-carcasses or quarters, and half carcasses into no more than three wholesale cuts, in slaughterhouses. Further cutting and boning must be carried out in a cutting plant”	Conventional methodology conserved by descriptive hygiene legislation. The text describes the actual number of cuts a carcass can be divided into. This does not affect hygiene.	The carcass and relevant parts must be presented for PM meat inspection so that a holistic examination can be undertaken and traceability upon and after approval maintained.	Alvseike et al. (2018)
(EU) 2019/627 of March 15, 2019. Section 3 Article 12 Point 4.	The speed of the slaughter line and the number of inspection staff present shall be such as to allow proper inspection.	Conventional methodology conserved by prescriptive hygiene legislation. “Slaughter line” describes the technical layout.	The processing speed, inspection area, adequate equipment and the number of inspection staff shall allow proper inspection of carcass or relevant parts and organs.	
(EU) 2019/627 of March 15, 2019. Section 3 Article 15 Point 2 and 3.	2. The official veterinarian shall require that carcasses of domestic solipeds, bovine animals over eight months old and domestic swine more than five weeks old are submitted for post-mortem inspection split lengthways into half carcasses down the spinal column. 3. If the post-mortem inspection so necessitates, the official veterinarian may require any head or any carcass to be split lengthways. However, to take account of particular eating habits, technological developments or specific sanitary situations, the official veterinarian may authorize the submission for post-mortem inspection of carcasses of domestic solipeds, bovine animals more than eight months old and domestic swine more than five weeks old that are not split in half.	Conventional methodology conserved by prescriptive hygiene legislation. Assumingly, the objective is to be able to inspect the spinal column. The text requires the carcasses split into halves to be able to inspect the spinal column. This normative formulation dictates how dressing and cutting of an animals' body is conducted.	Where inspection of the spinal column will aid in the outcome of PM inspection, the official veterinarian shall require that the spinal column of domestic solipeds, bovine animals over eight months old and domestic swine more than five weeks old is split lengthways.	Alvseike et al. (2018) Jackman and Hathaway (2012)
(EU) 2019/627 of March 15, 2019. Section 2 Article 11 Point 4.	4. Ante-mortem inspection shall include verification of food business operators' compliance with their obligation to ensure that animals have a clean hide, skin or fleece, to avoid any unacceptable risk of contamination of the fresh meat during slaughter.	Conventional methodology conserved by prescriptive hygiene legislation. It is impossible for the FBOs to ensure that animals have a clean hide, skin or fleece. nor are generally criteria established. The subjective judgement of cleanliness by personnel can vary and possibly induce unfair trade. It has been shown that dirty animals can be dressed hygienically.	AM inspection shall include verification of FBOs' compliance with the condition and cleanliness of animals in the lairage, so that the results meet objective functional hygiene and animal welfare demands. Gap: The level of cleanliness of hide, skin or fleece is not specified Gap: Heavily contaminated hide, skin and fleece's impact on animal welfare is not addressed	(Bosilevac, Nou, Osborn, Allen, & Koohmaraie, 2005) (Hauge et al., 2011) Hauge et al. (2012) (D Antic et al., 2010) McEvoy et al. (2000) Brown et al. (2000)

**Table 3:** Results from text analysis of New Zealand legislation with detection of normative formulations possibly hampering innovative approaches in the handling of food producing animals from lairage to processing a carcass into edible parts.

Paragraph ID	Paragraph text	Identified normative formulation	Suggested new formulation	References
Animal Products Notice Specifications for Products Intended for Human Consumption August 14, 2020 2.2 (1) (a-e not included).	The operator must ensure that premises or place, facilities, equipment and internal structures …. . is: a) impervious, non-absorbent and free from depressions, pits, cracks, and crevices that may harbour contaminants; b) easily cleaned and sanitized; c) unaffected by a corrosive substance with which it is likely to come into contact, to the extent necessary to ensure that it will not harbour contaminants and is not a source of contamination; d) durable, resistant to fracture, and capable of withstanding repeated exposure to normal cleaning and sanitising; e) in the case of surfaces (other than those used for walking or standing on during operations), are smooth and minimise the accumulation of condensation; f) in the case of material lining the walls, floors and ceiling, are of a colour that does not disguise contaminants having regard to the lighting arrangements.	Conventional methodology conserved by prescriptive hygiene legislation. There are two dimensions to this requirement, sanitary design and sanitary operations. The requirement is a mix of objective and subjective demands. Some criteria are not defined, e.g.: b), d), e), f) Some criteria are impossible to meet: a) Good intentions and recommendations but do not provide functional demands. It should not be up to the CA concern to decide what operations must be easy. Whether easy or not, it is the results that matters.	Sanitary design must a) make sure any material is not leaching foreign material into product. b) have a colour that allows visual identification of contaminants. c) provide sufficient light for each task (luminance). Sanitary operations must d) fulfil objective functional hygiene demands e.g., visual contamination and microbiological criteria. e) fulfil microbiological criteria after cleaning of premises.	Giske (2020) European Hygienic Engineering and Design Group (2018) Piccoli, Soci, Zambelli, and Pisaniello (2004) Brown et al. (2000)
Animal Products Notice Specifications for Products Intended for Human Consumption August 14, 2020 18.10 (1)	The operator must ensure that slaughter is carried out without unnecessary delay and in a way that manages the distribution and proliferation of contaminants of the carcass.	Conventional methodology conserved by prescriptive hygiene legislation. The logistics of the slaughter operation is the FBO's responsibility. The phrase is not addressing the hygiene directly neither objective nor measurable.	The operator must ensure that, following the dressing of an animal, the meat complies with objective functional hygiene demands e.g., criteria for microbiological and visual contamination.	Gill et al. (1978) Brown et al. (2000)
Animal Products Notice Specifications for Products Intended for Human Consumption August 14, 2020 18.10 (2)	The operator must ensure that slaughter is only performed at a rate at which bodies of animals can be accepted for dressing.	Conventional methodology conserved by prescriptive hygiene legislation. The logistics of the slaughter operation is the FBO's responsibility. The phrase is not addressing the hygiene directly neither objective nor measurable.	Delete sentence from paragraph	
Animal Products Notice Specifications for Products Intended for Human Consumption August 14, 2020 18.12 (2)	The operator must ensure that hygienic techniques are used during dressing and animals are not dressed on the floor	Conventional methodology conserved by prescriptive hygiene legislation. “Dressing on the floor” is a normative phrase, not an outcome, and should be deleted.	The operator must ensure that edible parts of a carcass comply with objective functional hygiene demands, e.g., criteria for microbiological and visual contamination.	Van der Merwe, Hoffman, Jooste, and Calitz (2013 (Brown et al., 2000)
Animal Products Notice Specifications for Products Intended for Human Consumption August 14, 2020 18.12 (3)	The operator must ensure, for farmed mammals and live possums, opening cuts and the process of hide and pelt removal and disposal is carried out in a manner that manages the contamination of the carcass from the hide or pelt. Hide roll back, as applicable, must be managed. The technique used must take into account the consistency of the faecal material associated with the type of animal material.	Conventional methodology conserved by prescriptive hygiene legislation. “Must be managed” is not objective. Specifically highlighting consistency of faeces is normative in the sense that it presupposes a dressing method. The FBO need to consider all relevant pathways for contamination as relevant to their process.	Operators must assess processing and identify contamination pathways to establish a dressing process where cross contamination from hide, internal organs, or other animals does not exceed objective functional hygiene demands e.g., criteria for microbiological and visual contamination.	Gill et al. (1976) Brown et al. (2000)
Animal Products Notice Specifications for Products Intended for Human Consumption August 14, 2020 18.12 (9)	The operator must ensure that contact between carcasses within the primary processing premises, prior to passing the post-mortem examination, where applicable, is minimised to the extent necessary to ensure that the potential spread of contaminants is minimised.	A normative expectation that FBOs cannot meet. Minimised is a vision, but too high ambition and in practice impossible Current important meat borne risks are not controlled by meat inspection. As such there is no risk-based reason to distinguish contact before and after meat inspection.	Delete from paragraph	Hdaifeh et al. (2020) (Animalia & Nortura, 2017)
Animal Products Notice Specifications for Products Intended for Human Consumption August 14, 2020 18.12 (11)	The operator must ensure that handling and processing procedures are carried out without unnecessary delay and in a manner that minimises the transfer, proliferation and redistribution of contaminants on and between animal material and animal product.	Conventional methodology conserved by prescriptive hygiene legislation. Unnecessary delay is not objective nor measurable. Minimised is a vision, but too high ambition and in practise impossible. Without objective criteria FBOs cannot predict what is acceptable. The phrase relies on qualitative assessments and may lead to unequal demands and unfair competition.	The operator must ensure that edible parts of a carcass comply with objective functional hygiene demands, e.g., criteria for microbiological, visual contamination and not damaged due to gas production in intestines, bacterial growth through intestines, deviating characteristics or rigor mortis can occur.	Brown et al. (2000)
Animal Products Notice Specifications for Products Intended for Human Consumption August 14, 2020 21.4 (1)	The operator must ensure that green offal from farmed mammals that is saved when inherent contamination is present is kept separate from any animal material or animal product intended for human consumption during its handling, processing and transportation until it: a) has been cleaned so that there are no visible contaminants; and b) is acceptably free of parasites, parasitic lesions, and foreign bodies.	Conventional methodology conserved by prescriptive hygiene legislation. “Acceptably free” is not objective and measurable. An assessment is made by CA and anything that has passed that assessment meet “acceptably free”. Thus b) can be deleted.	Delete b) from paragraph	
Animal Products Notice Specifications for Products Intended for Human Consumption August 14, 2020 21.4 (6)	The operator must ensure that: a) contamination of blood is minimised; and b) blood does not come in contact with the outer surface of any slaughtered animal	A normative expectation that FBOs cannot meet. Minimised is a vision, but too high ambition and in practise impossible. The requirement for collected blood in b) is normative based on conventional approach using hollow knives instead of the outcome required.	Blood intended for human consumption must comply with objective functional hygiene demands, e.g., criteria for microbiological and visual contamination.	Brown et al. (2000)
Animal Products Notice: AM and Post-mortem examination of Mammals, Ostrich and Emu Intended for Human Consumption. Clause 2.2 (1)	Operators must ensure that prior to slaughter, all animals undergo an ante-mortem examination in accordance with subclause (2) and (3) to assess suitability for slaughter.	A normative expectation that FBOs cannot meet. Impossible expectations to the assessors. AM examination is not defined, nor does any harmonised objective criteria exist. Significant differences have been documented between assessors. “Examination” suggests someone must physically examine the animals. This should be an assessment, which can be based on other factors than viewing animals if that is appropriate given FCI, country animal health status and similar. The animals often rest or sleep and should probably not be stressed for an AM examination.	Operators must ensure that prior to slaughter, all animals undergo an AM *assessment* to *determine* the suitability for slaughter based on objective functional demands for animal disease, hygiene and animal welfare. Gap: Standardised and objective methodologies needed.	Almqvist, Berg, and Hultgren (2021)
Animal Products Notice: Ante-mortem and Post-mortem examination of Mammals, Ostrich and Emu Intended for Human Consumption. Clause 2.2 (2)(a)	The examination under subclause (1) must be carried out by an ante-mortem examiner: within 24 h of arrival of the animals at the place of slaughter	Conventional approach conserved by descriptive hygiene legislation. Presumes animals arrive at the place of slaughter, and not the other way around.	The assessment under subclause (1) must be carried out within 24 h prior to slaughter.	Almqvist et al. (2021)
Animal Products Notice: Ante-mortem and Post-mortem examination of Mammals, Ostrich and Emu Intended for Human Consumption. Clause 2.7 (1)	A whole herd health scheme (WHHS) must contain the following particulars: i. the defined group or class of animals, not including bobby calves, the scheme relates to ii. a requirement that the defined group or class of animals be farmed, or managed, in accordance with the scheme, for not less than 6 weeks prior to being submitted for slaughter; iii. a requirement that no new animals are introduced into the defined group within 6 weeks prior to slaughter; iv. requirements for a unique animal identification system; v. procedures to ensure that the animals are under the care of a veterinarian appointed by the supplier of animal material; vi. a verifiable system for tracing the complete health status of all animals in the scheme; vii. a verifiable system for tracing all animal treatments administered to the animal covered by the scheme throughout its life; viii. procedures for checking animals for abnormalities prior to despatch to the slaughtering place; and ix. requirements for the keeping of appropriate records	Conventional methodology conserved by prescriptive hygiene legislation. While the requirements are descriptive, the outcome required is not objectively defined. Comment: WHHS means animals are exempt from AM examination	WHHS should ensure only healthy and appropriately identified animals are presented for slaughter. Animals must meet the FBO specified requirements for cleanliness. A whole herd health scheme (WHHS) must contain the following particulars: i. … ii. …	
Animal Products Notice: Ante-mortem and Post-mortem examination of Mammals, Ostrich and Emu Intended for Human Consumption. Clause 3.3 (1)	The operator must ensure that post-mortem examination is undertaken by a post-mortem examiner without delay following the dressing of an animal intended for human consumption and in accordance with the relevant risk management program and this Part.	Conventional methodology conserved by prescriptive hygiene legislation. The logistics of the slaughter operation is the FBO's responsibility. The phrase is not addressing the hygiene directly neither objective nor measurable. An objective maximum time should be used.	The operator must ensure that PM examination is undertaken by a PM examiner within 24 h following the dressing of an animal intended for human consumption.	EFSA (2020) (Gill et al., 1976)
Animal Products Notice: Ante-mortem and Post-mortem examination of Mammals, Ostrich and Emu Intended for Human Consumption. Clause 3.3 (2), (3) and (6)	(2) The post-mortem examiner must conduct post-mortem examination so as to minimise cross-contamination between carcasses and in accordance with the procedures relating to post-mortem inspection as described in the Post-mortem Examination Procedures. (3) The post-mortem examiner must conduct post-mortem examination so as to minimise cross contamination between carcasses and in accordance with the procedures relating to post-mortem inspection as described in the Post-mortem Examination Procedures.	A normative expectation that FBOs cannot meet. Minimised is a vision, but too high ambition and in practise impossible.	PM examinator must not handle the product beyond what is necessary to make a decision on its fitness for human consumption. PM examination must not compromise the hygiene so that criteria for microbiological and visual contamination is not met.	Brown et al. (2000)

**Table 4:** Results from text analysis of US legislation with detection of normative formulations possibly hampering innovative approaches in the handling of food producing animals from lairage to processing a carcass into edible parts.

Paragraph ID	Paragraph Text	Identified normative formulation	Suggested new formulation	Scientific ref
9 CFR 416.4 (b)	All food-contact surfaces, including food-contact surfaces of utensils and equipment, must be cleaned and sanitized as frequently as necessary to prevent the creation of insanitary conditions and the adulteration of product.	Conventional methodology conserved by prescriptive hygiene legislation. The phrases are ambiguous. “Clean and sanitize as frequently as necessary” is not objective. “Insanitary conditions” and “adulteration” are neither defined nor objective.	Equipment and utensils must maintain a hygienic status to prevent leaching foreign material into product. Sanitary conditions must fulfil objective functional hygiene demands e.g., visual contamination and microbiological criteria.	Giske (2020) European Hygienic Engineering and Design Group (2018) Brown et al. (2000)
9 CFR 416.3(a)	Equipment and utensils must be maintained in sanitary condition so as not to adulterate product.	Conventional methodology conserved by prescriptive hygiene legislation. The phrases are ambiguous and do not properly define what is consider sanitary conditions and how to actually maintain them.	Equipment and utensils must maintain a hygienic status so that product complies with functional hygiene demands e.g., visual contamination and microbiological criteria.	Giske (2020) European Hygienic Engineering and Design Group (2018) Brown et al. (2000)
9 CFR 354.221(b)	[Rooms for holding carcasses for further inspection] shall be equipped with locks and keys and the keys shall not leave the custody of the inspector in charge of the plant.	Conventional methodology conserved by prescriptive hygiene legislation. The phrase is normative. Facilities may use other ways than keys to secure the door.	The rooms for holding carcasses for further inspection shall only be accessible to authorised inspector in charge.	
9 CFR 310.18(a)	Carcasses, organs, and other parts shall be handled in a sanitary manner to prevent contamination.	A normative expectation that FBOs cannot meet. The phrase is an ambiguous statement and does not properly define how to handle in a sanitary manner	The FBO must ensure that edible parts of a carcass comply with objective functional hygiene demands, e.g., criteria for microbiological and visual contamination.	Brown et al. (2000)
9 CFR 416.2 (b2)	Walls, floors, and ceilings within establishments must be built of durable materials impervious to moisture and be cleaned and sanitized as necessary to prevent adulteration of product or the creation of insanitary conditions.	Conventional methodology conserved by prescriptive hygiene legislation. “Clean and sanitize as frequently as necessary” is not objective. “Insanitary conditions” and “adulteration” are neither defined nor objective.	Walls, floors, and ceilings must maintain a hygienic status to prevent leaching foreign material into product. Sanitary conditions must fulfil objective functional hygiene demands e.g., visual contamination and microbiological criteria.	Brown et al. (2000)
9 CFR 318.5 (d)	Heads for use in the preparation of meat food production shall be split and the bodies of the teeth, the turbinated and ethmoid bones, ear tubes, and horn butts removed, and the heads then thoroughly cleaned.	Conventional methodology conserved by prescriptive hygiene legislation. This is a prescriptive statement. While it clearly states the expected actions to take, it does not give an expected outcome or allow for a different method to ensure the head is sufficiently clean.	Heads for use in the preparation of meat must be cleaned to meet functional hygiene demands e.g., visual contamination and microbiological criteria.	

### 3.1 Text analysis of global guidelines and principles

Codex Alimentarius, FAO and WHO all give room for different systems for dressing of an animals’ body into a carcass with edible and inedible parts. Here, results are presented from the text analysis of Codex Alimentarius Codes with emphasis on formulations that possibly have an impact on innovative approaches. Identified formulations that can represent hurdles for innovation are listed in Table 1.

The Codex Alimentarius has some formulations that describe functional demands, and so facilitate changes in processing methods. Thus, the Codex Alimentarius General Principles of Food Hygiene CXC 1–1969 General Principles (i) states that “*Food safety and suitability should be controlled using a science-based preventive approach, for example, a food hygiene system. GHPs (Good Hygiene Practices) should ensure that food is produced and handled in an environment that minimise the presence of contaminants*.” Further in General Principles (v) it says that “*Control measures that are essential to achieve an acceptable level of food safety, should be scientifically validated*”. In General Principles (vii) it says “*Food hygiene systems should be reviewed to determine if modifications are needed. This should be done periodically and whenever there is a significant change that could impact the potential hazards and/or the control measures (*e.g.*, new process, new ingredient, new product, new equipment, new scientific knowledge) associated with the food business*.” This support implementing different innovative systems having a focus on the functional demands rather than normative descriptions imposing hurdles for innovation. “*Minimise the presence of contaminants*” is however a formulation that in practice is impossible to obtain. “*Reduce to an acceptable level*” would be a better phrase.

The Chapter 8–10 in Code of Hygiene Practice for Meat (CAC/RCP 58–2005) is the most relevant for this study due to the given scope. Subchapter 8.4 point 68 states that “*all areas and facilities where bodies of animals are dressed or meat may be present should be designed and constructed so that they facilitate GHP, and contamination of meat is minimised to the greatest extent practicable*” (Codex Alimentarius, 2005). This framework provides no hurdles for innovation and different systems can be applied and still be in concordance with the codes, although the aim is a vision without objective criterium.

Subchapter 9.2.3 states that *“Performance objectives or performance criteria for outcomes of process control systems act to allow maximum flexibility and technical innovation in the way the establishment operator achieves the required level of performance”* and further *“the competent authority should, whenever practicable, recognise different risk-based meat hygiene activities within its competence, which have been demonstrated to meet at least the same risk-based meat hygiene outcomes”*. These are formulations that are open to innovative approaches and refer to objective criteria. Good examples are the microbiological criteria in the EU, New Zealand and the USA (European Commission, 2005; FSANZ, 2016; USDA, 2001).

Point 128 in CAC/RCP 58–2005 states that *“establishment operators should meet the requirements of the competent authority in terms of presentation of edible parts of bodies of animals for post-mortem inspection. Parts of slaughtered animals that have been removed before post-mortem inspection is performed remain identifiable, as belonging to a single carcass (or group of carcasses) when required for post-mortem inspection”.* This formulation represents a functional demand that can be fulfilled by several systems. Further, paragraph 130 in CAC/RCP 58–2005 points out that “*the competent authority should encourage development and adoption of innovative technologies and procedures at the establishment level that reduce cross-contamination and enhance food safety*”. This formulation gives opportunities for developing and implementing innovative systems. A post-mortem inspection system should include “availability of inspection as soon as is practicable after completion of dressing” imposing demands that if a new system is implemented for example, the dressing of a carcass, the post-mortem inspection must also be facilitated.

### 3.2 Text analysis of EU legislation

Results from the text analysis of EU's preambles and legislation is presented in Table 2 with normative formulations possibly presenting hurdles for innovation.

Overall, the text formulations in 852/2004 Regulation (EC) No 852/2004 of the European Parliament and of the Council of April 29, 2004 on the hygiene of foodstuffs provides no hurdles for innovative approaches or systems. For this regulation, no normative formulations were identified in the text analysis.

Preambles to the Regulation (EC) No 853/2004 of the European Parliament and of the Council of April 29, 2004 laying down specific hygiene rules for food of animal origin states that *“flexibility is important to enable the continued use of traditional methods at any of the stages of production, processing or distribution of food and in relation to structural requirements for establishments”*. Further, the preambles describe how the Standing Committee on the Food Chain and Animal Health (European Parliament and Council, 2002) should contribute to resolving disagreement and preparing the EU commission to coordinate processes and take appropriate measures. The preambles describe the importance of scientific advice underpinning Community legislation on food hygiene, and that the European Food Safety Authority should be consulted whenever necessary.

### 3.3 Text analysis of New Zealand legislation

Results from the text analysis of the New Zealand legislation is presented in Table 3 with normative formulations possibly presenting hurdles for innovation. This does not include legislation that is issued specifically to meet the requirements of an importing country. Unlike the many other jurisdictions, the proportion of products that are produced to meet some importing country requirements varies by species but is about 95% for venison and beef, and 99% for sheep meat (New Zealand Government, 2020, pp. 1–115).

Generally, the domestic legislation does not provide a barrier to innovation. There are several issues specified below but largely they relate to ante-mortem and post-mortem inspection and several are the same wording or reference that is used in more than one place.

### 3.4 Text analysis of US legislation

Results from the text analysis of the US legislation is presented in Table 4 with normative formulations possibly presenting hurdles for innovation. Generally, the domestic legislation does not provide a barrier to innovation. There could be barriers based on interpretation that are presented in the guidance (directives) which were not included in this scope.

## 4 Discussion

The overarching objective of meat safety legislation is to protect human health. The Codex Alimentarius provides the global common ground for food safety systems, and interestingly these texts appear more focused on aims than methods. The review showed that text formulations used in meat safety regulations have many similarities in Europe, New Zealand and USA, and also share some challenges. The texts reflect the predominant slaughtering techniques with a large focus on slaughter lines and hygiene related to this form of slaughtering and meat processing. The tendency to interpret descriptive norms (i.e., what is) as prescriptive (i.e., what should be) is an early emerging bias to maintain the status quo (Roberts, 2021). These descriptions of today's state of the art provide context relevant for today's stakeholders but reduce flexibility which can impede industrial innovation and hygienic improvement i.e., normative phrases may preclude new technologies and disruptive changes.

### 4.1 Paradigm shifts and incremental improvements

**Fig. 2:**
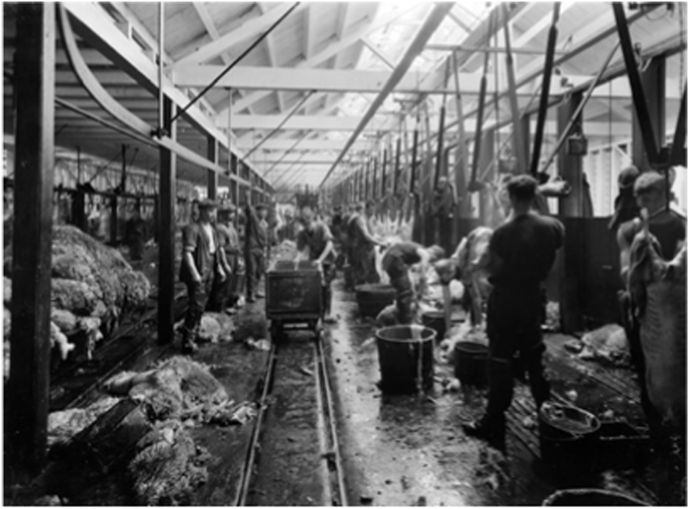
Processing sheep carcasses, Christchurch Meat Company. Webb, Steffano, 1880–1967: Collection of negatives. Ref: 1/1-019459-G. Alexander Turnbull Library, Wellington, New Zealand.

Innovations come in paradigm shifts (Kuhn, 1962) or as incremental improvements. Historically, domesticised animals were slaughtered on-farm by the farmers or ambulatory butchers when meat was in demand, and later, butchers were specialists and centralised slaughter in slaughterhouses. In modern times relatively few major principal changes to the slaughter process have taken place since the shift from solo butcher systems on a bench to slaughter in disassembly line systems. In New Zealand, this took place in the 1930s, not without significant disruption of the workforce (Inkson & Cammock, 1984). Since then, innovations in slaughter techniques have usually been incremental about different types of tools, machines and automation of single operations (Barbut, 2020; Clarke, Nielsen, & Madsen, 2014; Esper et al., 2021; Sigurdsson & Eiríksson, 2006). Thus, legislation favours conventional slaughtering performed in slaughter lines (Fig. 2).

All economic activities face expectations of productivity increase. The general industrial trend has been productivity increase first from increased volumes, then from specialization and finally from automation. However, an important share of food production is taking place in marginal regions where neither volume increase is possible, nor specialization is necessarily rational. Automation in the meat industry has been relatively slow because biological variation, the materials' heterogeneity, plasticity, wet and slippery surfaces, corrosive environment, and high hygienic standards together is the ultimate challenge. However, several completely new and automated carcass and meat processing solutions have been developed in recent years. Examples include the Mayekawa deboning system in Japan (Mayekawa MFG CO LTD, 2020), the Nolchiri intact carcass system in New Zealand (NolChiri Intact Meat Limited, 2018) and Scott Automation's automatic cutting system for lamb (Ruberg, 2021). In general, automated solutions has been tailored for the largest volume plants. The suggested Meat Factory Cell concept (MFC) addresses the future need for scalable, robust and flexible automation (Esper et al., 2021).

Assurance systems are crucial for securing the consumers safe meat, and therefore, strict and clear legislation is necessary. The legislation needs to develop along with new challenges (Sigurdsson & Eiríksson, 2006). Early forms of meat inspection had been around for centuries with examples in law from as early as 1706 (Brandly, Migaki, & Taylor, 1966). Meat inspection as we recognise it today, was introduced in the 1890's by Robert Ostertag and was based on visual inspection, palpation and incision of relevant organs (Ostertag, 1899). At that time, the meat inspection was risk-based to address the dominant hazards, but as time has moved on, the important zoonoses for public health has changed. Official veterinarians are in charge of meat inspections, and where and how this is done is regulated by law. These strict meat inspection procedures may therefore act as hurdles for innovation and more cost-efficient approaches, as food safety hazard panorama varies and changes regionally. The present legislation in Europe underlines the need for scientifically documented innovation. Slowly, the responses emerge, for example, simplified visual procedures omitting palpation of the lungs and the liver at meat inspection has been introduced in Denmark (Alban, Pacheco, Kruse, & Petersen, 2018), omitted tests based on epidemiological indicators regarding *Trichinella* in New Zealand (Eames, Raponi, Martinez, & Watts, 2021; Richardson, Cogger, Pomroy, Potter, & Morris, 2009) and *Taenia saginata* in Sweden (Livsmedelsverket, 2021b, pp. 1–40). New technologies have been implemented in a wider value chain approach to safe meat production as described in Meat Safety Assurance System (MSAS) (Blagojevic et al., 2021). Examples are spectroscopy in surveillance programmes for heavy metals and medical residuals (Gibson, Bratchell, & Roberts, 1988), serology (Engvall & Perlmann, 1971; Maganira, Kidima, Mwita, & Höglund, 2021; Nielsen et al., 2001), HACCP and verifying microbiological tests (Arvanitoyannis, 2009), programmes on *Salmonella* and *Campylobacter* (EFSA, 2011a; Sandberg, Ostensvik, Aunsmo, Skjerve, & Hofshagen, 2006) and polymerase chain reactions (PCR) (Mullis, Jose, Leath, Wennberg, & Joseph, 1997). In recent years, new enabling technologies have shown promising testing results in detecting invisible microbial contamination (Avershina et al., 2021; Burfoot, Tinker, Thorn, & Howell, 2011; Lee et al., 2010; Taxt, Avershina, Frye, Naseer, & Ahmad, 2020). Food Chain Information (FCI) has been implemented in EU legislation despite sparse documentation in advance but is expected to improve and assist in meat inspection (Blagojevic et al., 2021). In Norway, the meat sector has developed an electronic solution that includes FCI (Animalia, 2021) and is paralleled with the CA's meat inspection system, called MAKKS. VetInspector (IHFood AS, Copenhagen, Denmark) performs an automatic poultry meat inspection based on vision-technology combined with artificial intelligence (Jørgensen, Fagertun, & Moeslund, 2017). Similar approach has been invented for surveillance of lung lesions in pigs from *Actinobacillus pleuropneumoniae* and *Mycoplasma* spp. (Bonicelli et al., 2021; Trachtman et al., 2020). These examples represent the beginning of the digital transformation. Digital technologies will not only exchange analogue technologies, but alter the way we work, stakeholders' natural roles, and division of labour (Zaoui & Souissi, 2020).

Other technical improvements over the last few decades are decontamination methods of carcasses during slaughter, dressing and chilling. A variety of decontamination technologies have been developed to compensate for imperfect slaughter hygiene and the emergence of enterohaemorrhagic *Escherichia coli* infections (Dorsa, 1997; Hauge, Nafstad, Røtterud, & Nesbakken, 2012; Røssvoll et al., 2015). The use of chemical and physical interventions has been controversial and impeded by legislation in many countries for example, in Europe. The USA has been leading the development of decontamination systems, especially the use of hot water rinses and different chemical solutions to reduce microbial load on carcasses (Kochevar, Sofos, LeValley, & Smith, 1997; Sofos et al., 2000).

In recent years, the use of novel, non-thermal processing technologies such as pulsed electric fields (PEF), high-pressure processing (HPP), shockwave, ohmic heating and ultrasounds, have experienced extensive development. They can assist the industry in achieving higher safety and quality standards by reducing microbial loads and modifying meat properties (Hugas, Garriga, & Monfort, 2002). Recent studies have for instance shown that HPP to early post-mortem meat, can maximise the water holding capacity and tenderness of the meat (Bolumar et al., 2021). However, HPP applied on hot boned primal cuts could affect meat inspection and carcass grading if those procedures are supposed to happen subsequently on a slaughterline. Another example is the utilization of UV radiation in the production of fresh meat, whose use is prohibited in the legislation (EU) 2019/627 Chapter III Article 45: *Meat is to be declared unfit for human consumption if it: l) has been treated illegally with ionising or UV-rays* (European Commission, 2019b). The main concern is that such decontamination technologies can mask unhygienic slaughter and dressing (Hugas & Tsigarida, 2008), but concomitantly, technologies that can increase food safety cost-efficiently are discarded. Interestingly, similar set of arguments were discussed before introduction of milk pasteurisation before World War II.

### 4.2 Legislative text phrases

What humans perceive as normal, has been found to be influenced both by what they believed to be descriptively average and by what they believed to be prescriptively ideal, with a general tendency for the normal to be intermediate between average and ideal (Bear & Knobe, 2017). This parallels nicely with research in psychology where it has been found that children infer what ought to be the case (prescriptive) after simply learning what is (descriptive) the case (Roberts, Gelman, & Ho, 2017). When making laws, normative formulations, descriptive or prescriptive, used in many law texts, may be a way for legislators to put their compound goals into a familiar context. The lawmakers’ experiences form the mental models of how things are or should be. Being too instructive, the texts may conserve outdated practices and structures. Prescriptive concerns should be adopted to advance specified ends (Jolls, Sunstein, & Thaler, 1998). In EU legislation, the preambles of the regulation 2017/625 point 45, already signal the ability to develop official control (meat inspection) along with innovation and new techniques without risk to human health.

One of the main difficulties with perceiving legislation for all stakeholders is voluminous texts. Normative phrases and excessive contextual parts increase volume considerably and may introduce contradictory requirements. Shorter legislative texts are appreciated by both FBOs and CAs, but there is also a need for legislative text that are understandable and practically applicable. This could preferably be addressed with guidelines, vademecums, standards, textbooks, research papers, etc.

Companies that are exporting meat globally experience different requirements and specifications in different markets (New Zealand Government, 2021, pp. 1–40). If these are objective measurable functional demands, the exporters can assess themselves the opportunities for business. If the different jurisdictions apply different normative phrases, *trans*-jurisdictional trade are formally difficult.

### 4.3 Science-based or politics

Another general finding in this review study, were the numerous demands that are difficult or impossible to obtain. In traditional meat production, those phrases are only visions, for example, “clean”, “avoid”, “minimise” and “maximise”. Minimise means to bring something to a minimum, and with decontamination or sterilising technologies it is not a problem to remove all vegetative hazards and spores. However, FBOs can optimise the production to meet the microbiological criteria for invisible contamination (Brown et al., 2000) or reduce and remove visible contamination to an acceptable level. The problem is that these visions in a legislative text become legislative demands. Cultural, political and economic differences result in different regional interpretations of what these phrases demand. The CA are in power to determine the meaning. It is the authors’ impression that the result in practice is continuous discussions and negotiations on plant and sometimes useless investments in technologies that do not work better. This is neither more effective regarding food safety nor cost-efficient.

### 4.4 Risk-based meat inspection and flexibility

Developing risk-based meat inspection has been discussed for decades (Nordisk ministerråd, 2007; Batz et al., 2005; Berends & van Knapen, 1999). The EU Council invited the EU Commission to maintain the benefits of simplification and flexibility, and prepare concrete proposals allowing the effective implementation of modernised sanitary inspection in slaughterhouses while making full use of the principle of the risk-based approach (Council of the EU, 2009). The principle of a risk-based approach is that limited resources shall be spent on activities that produce the safest food, highest animal welfare and animal health per monetary unit. Also, food wholesomeness and sustainable production have gained increasing importance in recent years and challenge how these different dimensions of meat production should be balanced optimally in the future (Blagojevic et al., 2021). As the prevalence of hazards varies considerably between regions (EFSA, 2011b), it is impossible to apply a risk-based approach without a certain degree of flexibility. Blanket policies and control actions for regionally eradicated diseases are not risk-based. It is neither effective, cost-efficient nor fair competition, if countries and regions with animal husbandry systems and effective disease prevention systems, run the same control activities as regions where the hazards’ prevalence cause significant risks to public health. Cost-efficient surveillance and transparent documentation are also indicated in regions with favourable epidemiology.

### 4.5 Functional demands and objective criteria

EFSA and ECDC have been asked to define animal and human health epidemiological criteria required to make the EU member states able to carry out risk analysis, if appropriate, to adapt the general inspection methods within the framework provided by the legislation (EFSA, 2013) based on the trends and sources of zoonoses, zoonotic agents and microbiological resistance in the EU, and harmonised monitoring of food-borne infections (EFSA, 2011c).

Prevalence and incidence of hazards in animals, meat or consumers have been suggested as Harmonised Epidemiological Indicators (HEI) and that these could be used to adapt meat inspection methods (EFSA, 2011b). Functional demands include HEI criteria but also include criteria beyond HEIs, for example, hygiene indicators, visible contamination, and criteria for acceptable animal welfare and technical performance of equipment, critical control points included. “Functional” underlines that a demand should achieve an acceptable level or standard that works. Hence a functional demand must be measurable against objective criteria. Food microbiology, which often only measure a proxy of a hazard, is hampered with considerable sources for variation and therefore standard methodology from sampling, laboratory analyses, statistical analyses and reports are needed (EFSA, 2011c). Unfortunately, there are a wide variety of methods applied that have not been validated. Then the results get unnecessary spurious, an issue that sometimes seems neglected by CAs (Alvseike et al., 2019; Røssvoll et al., 2017, 2017). Baseline studies are very useful to align the results across national and international borders. They are based on strictly standardised methodology and have been undertaken in many jurisdictions (Bohaychuk, Checkley, Gensler, & Barrios, 2009; EFSA, 2008; Phillips, Jordan, Morris, Jenson, & Sumner, 2006, 2012; USDA & FSIS, 2011). Still, even with the same methodology over time applied in a single abattoir, variation of bacteriological counts is a weakness.

Risk assessments are still mostly an academic and governmental subject. The link from functional demands for the FBOs to HEIs on human health epidemiological criteria is not in daily use by FBOs nor the CAs. The missing link is mainly the governments’ reluctance to express risk management decisions as appropriate levels of protection (ALOPs), which includes budgeting disease and deaths. Covid-19 has brought these questions to surface, but still the different “public health budgets” are not expressed directly. In reality, different systems and structures in different countries result in report bias and incomparable incidence rates. It exemplifies how cultural and economic differences impact ALOP by different governments.

## 5 Conclusion

In this review, design and formulation of current red meat safety legislation, guidelines and principles have been assessed for hurdles for innovation including the process from lairage to dressing of an animals' body into edible parts. Relevant food safety legislation contains prescriptive or descriptive parts which may restrict innovative methods of meat production and processing. Legislation should offer conditional flexibility in design and implementation, presenting functional demands and the intended food safety outcomes within a risk management framework. This review suggests alternative text formulations providing better opportunities for innovation, which could be considered for legislative text improvements. Objective normative phrases in legislation can function as a least common multiple if alternative methods are allowed too on condition that they fulfil objective criteria. Competence and practical advice should be sought in white papers, FBO's own guidelines, expert consultancy or from suppliers. When meat safety legislation is amended in the future, functional demands and objective criteria should be the goal.

## Declaration of competing interests

The authors declare that they have no known competing financial or personal relationships that could have appeared to influence the work reported in this review paper.
